# k-t SENSE-accelerated myocardial perfusion MR imaging at 3.0 Tesla – comparison with pressure wire measurement of fractional flow reserve

**DOI:** 10.1186/1532-429X-11-S1-P46

**Published:** 2009-01-28

**Authors:** Tim Lockie, Divaka Perera, Simon Redwood, Sebastian Kozerke, Michael Marber, Eike Nagel, Sven Plein

**Affiliations:** 1 Rayne Institute, St Thomas' Hospital, London, UK; 2 ETH, Zurich, Swizerland & Rayne Institute, St Thomas' Hospital, London, UK; 3 Academic Unit of Cardiovascular Medicine, University of Leeds & Rayne Institute, St Thomas' Hospital, London, UK

**Keywords:** Fractional Flow Reserve, Coronary Territory, Fractional Flow Reserve Measurement, Pressure Wire, Gradient Echo Pulse Sequence

## Introduction

*k*-space and time sensitivity encoding (*k-t* SENSE) exploits coil encoding and spatiotemporal correlations and thus allows substantial acceleration of CMR data acquisition. *k-t* SENSE has been used to improve temporal or spatial resolution of perfusion CMR at 1.5 Tesla. In this prospective study high spatial resolution *k-t* SENSE CMR perfusion at 3 Tesla was compared to fractional flow reserve (FFR), as the reference method for detection of flow-limiting coronary stenoses in the catheter laboratory.

## Purpose

To determine the diagnostic accuracy of high spatial resolution *k-t* SENSE CMR perfusion at 3 Tesla to detect flow-limiting coronary stenoses in patients with stable coronary disease.

## Methods

*k-t* SENSE accelerated perfusion CMR was performed on a 3 Tesla Philips Achieva system. A saturation recovery gradient echo pulse sequence was implemented with a repetition time/echo time 3.0 ms/1.0 ms, flip angle 15°, 5× *k-t* SENSE acceleration, 11 interleaved training profiles, spatial resolution 1.1 × 1.1 × 10 mm^3^, 3 slices acquired at each RR interval. Patients with known or suspected coronary artery disease were studied during adenosine stress (an intravenous infusion of adenosine at 140 μg/kg/min, Adenoscan, Sanofi-Synthelabo, Guildford, United Kingdom) and at rest. Main exclusion criteria were previous MI, previous CABG, renal failure, severe LV dysfunction or significant co-morbidities. All patients underwent clinically indicated cardiac catheterisation within 48 hours of CMR. Invasive measurements of FFR were performed in the catheter laboratory on all lesions of more than 40% severity. FFR was calculated as (P_d_-P_v_)/(P_a_-P_v_), where P_a_, P_v_ and P_d_ are simultaneous aortic, right atrial and distal coronary pressures measured during an intravenous infusion of adenosine at 140 μg/kg/min. A vessel with <40% stenosis angiographically was assumed to be non-flow limiting. The ability of visual analysis of CMR to detect the presence of myocardial ischaemia per coronary territory was determined, blinded to the results of the angiogram and the FFR result.

## Results

**Figure 1:**
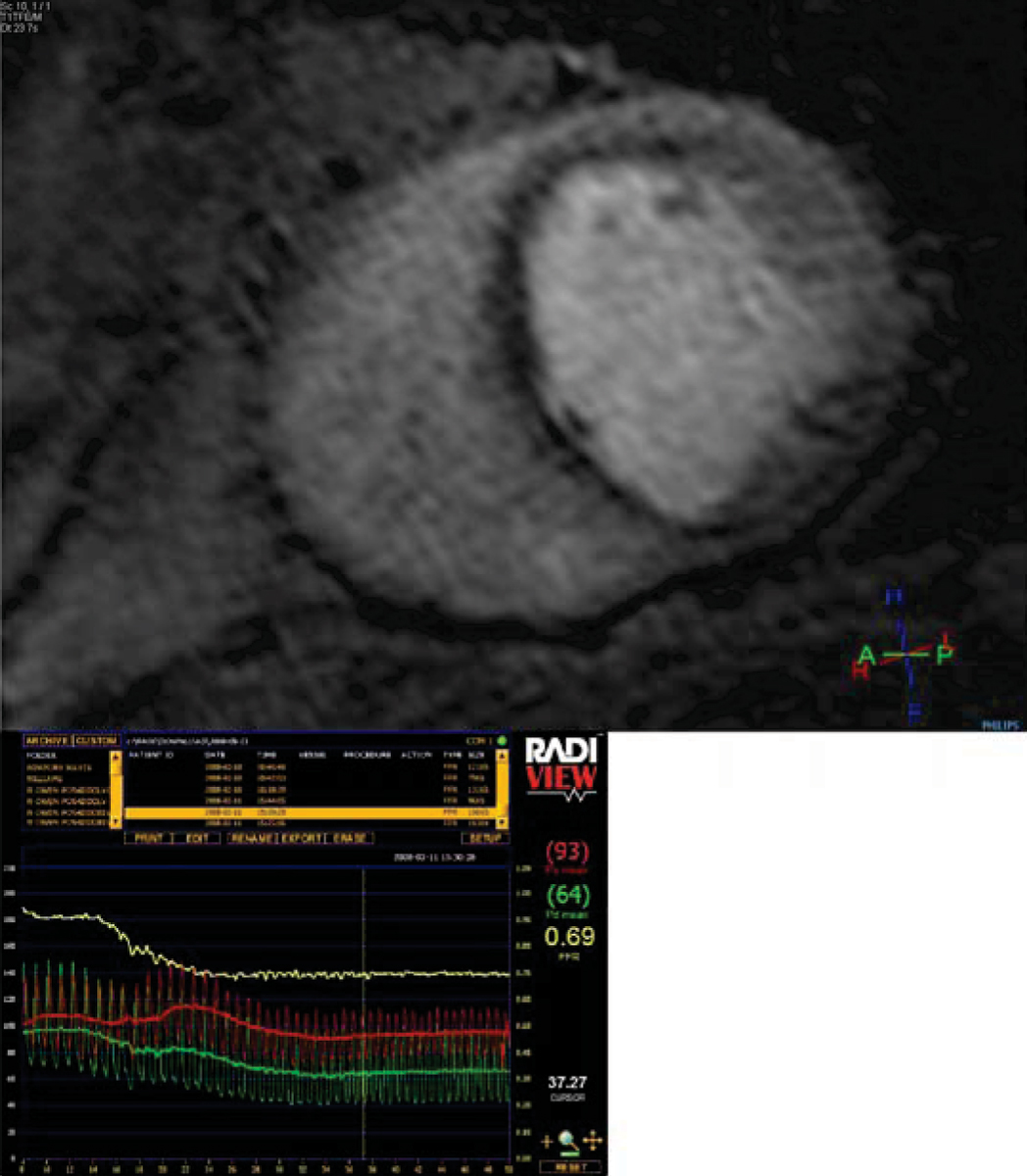
**An anterior wall perfusion defect with FFR trace below showing a significant lesion (FFR 0.69) in the proximal left anterior descending artery**. An FFR < 0.75 is considered to be haemodynamically significant.

Seventeen patients were recruited (12 male, age 64.1 ± 7.2 years). One patient was excluded from the analysis because of technical problems with the FFR measurement, so that 48 coronary territories were studied. There were no complications with the adenosine infusion in either the CMR scan or during the pressure wire studies. Mean scanning time was 54 ± 15 minutes. Coronary stenosis of >40% was seen in 20 vessels which underwent successful pressure wire assessment. Of these, 10 lesions had an FFR < 0.75 (mean 0.50 ± 0.14) and 10 lesions had an FFR ≥ 0.75 (mean 0.87 ± 0.94). Sensitivity and specificity of CMR perfusion to detect coronary stenoses at a threshold of FFR < 0.75 was 84% and 97%, respectively. An example of an anterior-septal wall perfusion defect with the corresponding FFR trace is shown in figure 1.

## Conclusion

*k-t* SENSE accelerated high-resolution perfusion MR at 3.0 Tesla accurately detects flow-limiting coronary artery disease as defined by FFR. Compared with previous reports, these results demonstrate very high specificity of perfusion CMR, possibly as a result of the high spatial resolution at which endocardial dark rim artefacts are reduced.

